# Strategies to uncover undiagnosed HIV infection among heterosexuals at high risk and link them to HIV care with high retention: a “seek, test, treat, and retain” study

**DOI:** 10.1186/s12889-015-1816-0

**Published:** 2015-05-10

**Authors:** Marya Gwadz, Charles M. Cleland, Holly Hagan, Samuel Jenness, Alexandra Kutnick, Noelle R. Leonard, Elizabeth Applegate, Amanda S. Ritchie, Angela Banfield, Mindy Belkin, Bridget Cross, Montserrat Del Olmo, Katharine Ha, Belkis Y. Martinez, Talaya McCright-Gill, Quentin L. Swain, David C. Perlman, Ann E. Kurth, the BCAP Collaborative Research Team

**Affiliations:** NYU College of Nursing, Center for Drug Use and HIV Research (CDUHR), 433 First Avenue, 7th Floor, New York, NY 10010 USA; Department of Epidemiology, University of Washington, Box 357236, Seattle, WA 98195 USA; Mount Sinai Beth Israel, Baron Edmond de Rothschild Chemical Dependency Institute, 120 Water Street, Floor 24, New York, NY 10038 USA

**Keywords:** Protocol, Health status disparities, HIV, Undiagnosed HIV, Engagement in care, Venue-based sampling, Respondent-driven sampling, Peer-driven intervention, African American, Latino

## Abstract

**Background:**

Over 50,000 individuals become infected with HIV annually in the U.S., and over a quarter of HIV infected individuals are heterosexuals. Undiagnosed HIV infection, as well as a lack of retention in care among those diagnosed, are both primary factors contributing to ongoing HIV incidence. Further, there are racial/ethnic disparities in undiagnosed HIV and engagement in care, with African Americans/Blacks and Latinos remaining undiagnosed longer and less engaged in care than Whites, signaling the need for culturally targeted intervention approaches to seek and test those with undiagnosed HIV infection, and link them to care with high retention.

**Methods/Design:**

The study has two components: one to seek out and test heterosexuals at high risk for HIV infection, and another to link those found infected to HIV care with high retention. We will recruit sexually active African American/Black and Latino adults who have opposite sex partners, negative or unknown HIV status, and reside in locations with high poverty and HIV prevalence. The “Seek and Test” component will compare the efficacy and cost effectiveness of two strategies to uncover undiagnosed HIV infection: venue-based sampling and respondent-driven sampling (RDS). Among those recruited by RDS and found to have HIV infection, a “Treat and Retain” component will assess the efficacy of a peer-driven intervention compared to a control arm with respect to time to an HIV care appointment and health indicators using a cluster randomized controlled trial design to minimize contamination. RDS initial seeds will be randomly assigned to the intervention or control arm at a 1:1 ratio and all recruits will be assigned to the same arm as the recruiter. Participants will be followed for 12 months with outcomes assessed using medical records and biomarkers, such as HIV viral load.

**Discussion:**

Heterosexuals do not test for HIV as frequently as and are diagnosed later than other risk groups. The study has the potential to contribute an efficient, innovative, and sustainable multi-level recruitment approach and intervention to the HIV prevention portfolio. Because the majority of heterosexuals at high risk are African American/Black or Latino, the study has great potential to reduce racial/ethnic disparities in HIV/AIDS.

**Trial registration:**

ClinicalTrials.gov, NCT01607541, Registered May 23, 2012.

## Background

Despite advances in antiretroviral treatment, prevention strategies, and HIV diagnostic testing technologies, HIV disease incidence in the United States has remained steady, with over 50,000 individuals becoming infected annually [1]. Undiagnosed HIV infection is a major driver of these incident infections, although only 18 % of infected persons nationally are unaware of their HIV status [2–4]. In fact, 44 to 66 % of new HIV infections are attributed to this modest proportion of individuals with undiagnosed HIV [3, 5]. Moreover, there are serious racial/ethnic disparities in undiagnosed HIV, with African Americans/Blacks and Latinos remaining undiagnosed longer than Whites [6]. Further, undiagnosed HIV is more common in males compared to females, and among heterosexual males compared to men who have sex with men (MSM) [6]. (Acronyms used in this protocol description are defined in Table 1.)

**Table 1:** Acronyms used

ACASI	Audio Computer-Assisted Interviewing format
ART	Antiretroviral therapy
BCAP	Brooklyn Community Action Project
CDC	Centers for Disease Control and Prevention
HHR	Heterosexuals at high risk (for HIV)
HRA	High-risk area
MSM	Men who have sex with men
NHBS	National HIV Behavioral Surveillance
PDI	Peer-driven intervention
PWID	Persons who inject drugs
RDS	Respondent-driven sampling
STTR	Seek, Test, Treat and Retain
VBS	Venue-based sampling
VDT	Venue, day-time units

In 2010, the National Institute on Drug Abuse (NIDA) at the National Institutes of Health called for research on new approaches to seek out persons with undiagnosed HIV, provide them with counseling and testing, and link those found to be HIV infected into medical care, with high retention, which are referred to as “Seek, Test, Treat, and Retain” (STTR) studies. This paper summarizes the study protocol for our STTR study funded under this initiative, which is being conducted in the borough of Brooklyn in New York City, called the “Brooklyn Community Action Project” (BCAP).

## High-risk heterosexuals in the U.S

The National HIV Behavioral Surveillance (NHBS) system estimates the prevalence of HIV infection in three high-risk populations on a rotating basis in 20 Metropolitan Statistical Areas across the U.S.: heterosexuals at high risk (HHR), men who have sex with men (MSM), and persons who inject drugs (PWID). The NHBS studies use two main sampling frames across study locations, as determined by the CDC: venue-based sampling (VBS), generally used for the MSM studies, and respondent-driven sampling (RDS), used to enroll PWID and HHR [7]. However, VBS and RDS have never been directly compared in the same Metropolitan Statistical Area in terms of recruiting populations who may benefit from STTR interventions.

Nationally, over 25 % of incident HIV infections have been attributed to heterosexual activity [8]. In 2010, the national NHBS with HHR found an estimated HIV prevalence of 2.3 % [8]. The NHBS study design for HHR targets adults who are sexually active with opposite sex partners and who are socially and geographically connected to areas with high HIV prevalence, which also happen to be high-poverty areas, in which African American/Black and Latino persons are the predominant race/ethnicity [9, 10]. Indeed, the HIV disease burden in these communities is much higher in these geographical areas than across the U.S. generally, where the overall HIV prevalence is estimated at 0.6 % [11]. Locally, in the 2006 New York City NHBS study of HHR, for which detailed data have been published, HIV prevalence was 8.6 %, with an estimated HIV incidence in this population of 3.31 % per year and 2.59 % per year among those with no history of drug injection or male-to-male sex [12]. Further, Brooklyn had the highest HIV prevalence and the highest rates of risk factors (unpublished observations, Holly Hagan, 2010), and is therefore the geographic focus of the present study.

Yet HHR are understudied relative to other HIV risk groups [13]. One difficulty in establishing research priorities for HHR has been the lack of a universal definition of the population. Heterosexual activity alone is overly broad to define a high-risk subgroup, while the CDC surveillance definition of documented heterosexual contact with someone known to be HIV-infected is too narrow [1, 12]. The NHBS definition of HHR is persons with sexual partners of the opposite sex linked within urban geographical areas with high rates of heterosexually transmitted HIV, which also have high rates of poverty [14]; these geographical locations are called “high-risk areas” (HRAs). Due to the marked over-representation of African American/Black and Latino populations in these high prevalence/high-poverty neighborhoods, the majority of HHR are from these two racial/ethnic groups [9]. Thus the CDC has called for research to test culturally appropriate interventions to overcome barriers to HIV testing and increase linkage to HIV care for heterosexuals in high-risk urban areas [8].

## Testing, linkage, and retention for HHR

Diagnostic HIV testing is critical to HIV prevention, and the CDC recommends annual testing for vulnerable populations such as HHR [14]. Yet rates of regular HIV testing are inadequate in this group, and as a result, late diagnosis of HIV is common [15, 16]. A recent local NHBS study found that while over 90 % of HHR had encountered at least one setting where HIV testing is offered in the past year, only a third had actually been tested, suggesting that HHR often decline testing when it is offered [15]. Moreover, vulnerable subgroups of HHR encounter settings where testing is offered infrequently [17]. Thus culturally acceptable and efficient active approaches to seek out HHR and engage them in high-quality HIV testing are needed.

Post diagnosis, there are a number of serious gaps in HIV care engagement, and clinical and health outcomes [18, 19]. Of the 1.1 million Americans living with HIV, 60 % are not retained in care; 63 % have not been prescribed antiretroviral therapy (ART); and only 30 % have undetectable HIV viral load levels, the latter being the ultimate goal of HIV treatment [20]. The CDC has called for improvement in outcomes across this HIV care continuum, with particular efforts to reduce racial/ethnic disparities [21], since African Americans/Blacks and Latinos show lower rates of engagement in every indicator. Moreover, by risk group, HHR may experience longer delays before entering HIV care compared to MSM.

## Barriers to HIV testing and timely engagement in HIV care for HHR

In our study, we conceptualized the multi-level barriers that African American/Black and Latino HHR experience to HIV testing and engagement in HIV care within the Theory of Triadic Influence [22]. This social cognitive theory emphasizes three “streams of influence” on health behavior: the individual/attitudinal, the social, and the structural.

At the individual/attitudinal level of influence, barriers to testing for HHR include lack of awareness of recommended testing frequency, and low perceived risk of HIV infection stemming from beliefs that HIV affects mainly PWID and MSM [23, 24]. At the same time, fear of HIV testing and of the consequences of a positive test result are common powerful barriers [25, 26], along with mistrust of medical environments [25]. Substance use, which is common among HHR, has also been shown to serve as a barrier [25, 27]. Moreover, the population has other competing priorities, complicated by low socioeconomic status, such as mental health problems, unstable housing, and family and child-care responsibilities [17, 23]. At the social level, the potential stigma of a positive test result serves as a barrier to HIV testing [28]. Peer norms regarding health care, including that regular HIV testing is not necessary for HHR, may impede testing [29]. At the structural level, HHR have poorer access to settings where high-quality HIV testing is offered [15, 30]. Theoretically, barriers at these three levels of influence interact to impede access to HIV testing and reduce motivation to test among HHR. Furthermore, this same set of barriers also impedes linkage to and retention in HIV primary care among HHR diagnosed with HIV [31–33]. The present protocol describes two culturally targeted STTR intervention strategies for HHR to reduce these barriers to HIV testing and timely engagement in HIV care.

## Peer-driven intervention and respondent-driven sampling

Our study features a peer-driven intervention (PDI), in which participants are educated by peers on a set of core messages, then engage in structured facilitated sessions, followed by the opportunity to educate their own peers [34, 35]. The core messages target the specific barriers to the health outcome of interest - HIV testing and engagement in HIV care in this case - and are repeated and expanded upon throughout the intervention activities. Peers who receive the education from study participants are also invited to join the research study, and during the course of the intervention, they are trained to educate their own peers. (The BCAP intervention manual is available from the first author).

Because PDI involves peer-to-peer contact, it can serve as the basis for a sampling methodology for studying subpopulations that are hard to define, reach, and/or engage: respondent-driven sampling (RDS). RDS is network-based method for recruitment, similar to traditional snowball sampling, but with the goal of minimizing biases typically associated with those traditional methods [36, 37]. In RDS, a modest number of individuals are recruited directly by project staff (called “initial seeds”), and then trained to recruit a small number of their peers into the study. These peers then enter the study and peer recruitment continues until the sample size goals are met [36, 38]. The RDS method has four essential elements: tracking of recruitment chains; rationing of recruitment (usually 2-5 peers each); information on personal networks must be gathered (network size, recruitment refusals); and recruiters and recruits must have a pre-existing relationship [39].

## Aims

**Fig. 1:**
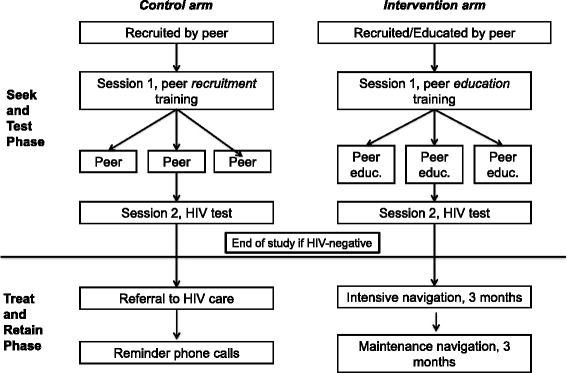
Two-phase BCAP PDI/RDS Model

The present study has two phases, a Seek and Test Phase and a Treat and Retain Phase. As shown in Fig. 1, the PDI/RDS study component includes activities to seek out and test HHR and strategies to link those found to be HIV-infected to medical care in a timely fashion. However, by design, we do not hypothesize differences in acceptance of HIV testing, which is > 90 % in similar research studies [12, 14, 40, 41], or in the prevalence of undiagnosed HIV between intervention and control arms, although these will be documented in the Seek and Test Phase. Thus the main aim of the study is to measure the efficacy of the BCAP PDI compared to a time- and attention-matched control intervention on the second-phase Treat and Retain outcomes, including 1) time to an HIV clinic appointment; 2) time to initiating ART if indicated; 3) HIV viral load suppression; and 4) retention in care among the newly diagnosed. These outcomes are assessed with serology (viral load suppression) and medical record abstraction. We hypothesize the PDI arm, compared to the control arm, will produce a shorter time to care entry and ART initiation, higher viral load suppression, and superior retention in care. The study also will project the clinical impact, costs, and cost-effectiveness of these two strategies (PDI vs. control) for care linkage and retention. Thus although the Seek and Test Phase temporally precedes the Treat and Retain Phase, we begin by describing the Treat and Retain phase, because it is the primary focus of the present study.

## Part 1: Treat and retain phase

### Methods

#### Study setting

In the planning stage of the study in 2011, a core high-risk area (HRA) was defined within Brooklyn, the borough in New York City with the highest heterosexual HIV prevalence in the 2006 NHBS study, which were the most up-to-date available NHBS data at the time. The classification of the HRA was based on the definition created in the NHBS studies in 2006, namely, a small set of zip codes with both the highest prevalence of poverty and rates of heterosexual HIV [14, 15]. We selected a new HRA cluster for the present study using updated HIV surveillance and 2011 demographic data, based on the approach described in the next section [42].

#### HRA index

Two inputs were obtained for all of the 52 Brooklyn zip codes: 1) the percent of households in poverty, based on 2009 projections from U.S. Census [43]; and 2) the number of HIV diagnoses from 2005 to 2009, from New York City Department of Health and Mental Hygiene case surveillance data [44]. For each zip code in Brooklyn, a numerical “HRA index” was calculated by standardizing the poverty and heterosexual HIV prevalence to the overall levels in Brooklyn. The HIV prevalence was calculated based on a denominator of adults in each zip code, assuming the fraction of non-heterosexually active adults was relatively small and evenly distributed across the geography. The final HRA index for each zip code was the sum of the standardized HIV and poverty scores.

##### Selecting the HRA cluster

**Fig. 2:**
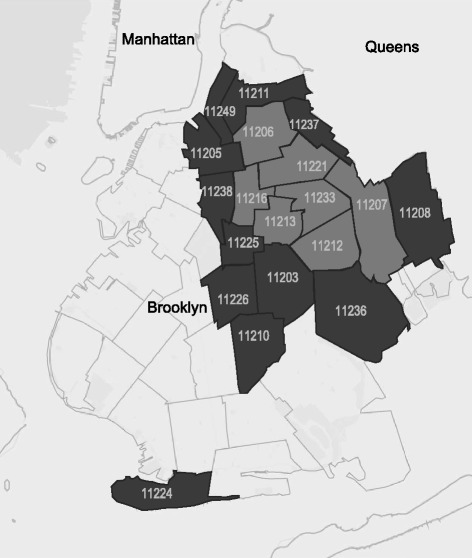
Core High-risk Area (HRA; in light grey) and surrounding larger HRA (dark grey), in the borough of Brooklyn

The 52 indexed zip codes were then geographically mapped to establish a threshold for the final HRA cluster selection. The goal was to select a set of zip codes based on a high index value that would form a geographic cluster corresponding to a contiguous set of neighborhoods. This cluster analysis was completed qualitatively and quantitatively. Qualitative assessments were made by examining maps with HRA thresholds from the top 10 to 40 % of index values. Quantitatively, we used a global spatial autocorrelation statistic (Moran’s I) to determine the extent of clustering, then a locally indicated spatial autocorrelation statistic (LISA Moran’s I) to quantify the location of any clustering [45]. Based on this analysis, the HRA for the present study was defined as the top 25 % of the zip codes (see Fig. 2, the light grey region), a contiguous block of 7 zip codes that included several neighborhoods in central Brooklyn. We refer to this region as the “core HRA”. Since HHR move freely across the boundaries of the core HRA, we also selected a lower threshold to reduce artificial restriction of RDS recruitment chains. This *larger HRA* comprised the top 50 % of the HRA index, corresponding to an additional 11 zip codes (see Fig. 2). The BCAP study field site was located in the *core HRA*, as was recruitment of “initial seeds” to start the RDS recruitment chains. However, recruitment of peers could extend to the larger HRA.

#### Study design using PDI/RDS

As noted above, RDS begins with direct recruitment of a small number of initial seeds who start recruitment chains by recruiting their peers into the research [36, 38]. In the present study, initial seeds are randomized to an intervention or control arm at a 1:1 ratio. Further, eligible and consenting peers who are subsequently recruited into the study are assigned to the same arm as their recruiter. Thus each initial seed and the peers subsequently enrolled in his/her recruitment chain are considered a cluster. This cluster randomized controlled trial design is necessary because intervention activities begin at the time of recruitment, when recruiters educate peer recruits on core messages. Because the statistical power of a clustered trial increases with the number of clusters, we plan to recruit more seeds than is typical in RDS (namely 40 seeds per arm), with a target sample size of 3000 individuals. The PDI/RDS study component also includes those with previously diagnosed infection (called “known positives”), and will describe HIV health indicators and engagement in HIV care among this subgroup. Study activities are approved by the Institutional Review Board at the NYU School of Medicine, and the protocol is registered with clinicaltrials.gov (NCT01607541).

#### Randomization

A list of random allocations for initial seed participants is generated by the study statistician in randomly ordered permuted blocks of 2, 4, 6, 8, or 12 allocations. The allocation list is stored in a password-protected file that can only be accessed by the study statistician, data manager, and project director. None of these individuals are responsible for consent, enrollment, or delivery of intervention content to participants. After informed consent is obtained, field staff will contact the statistician, data manager, or project director by phone for an allocation for each initial seed. During this phone call, the statistician, data manager, or project director records the unique identification number of the initial seed and the date on the allocation list, and then relays the assigned study arm to staff in the field.

#### The BCAP PDI

Motivational Interviewing is the intervention’s main counseling approach. Motivational Interviewing is a flexible, collaborative counseling method that actively engages, focuses, and guides participants, without judgment or pressure, in order to elicit and strengthen durable, high quality intrinsic motivation for behavior change [46]. The BCAP PDI is culturally targeted to address the main barriers that African American/Black and Latino HHR experience to HIV testing and linkage to HIV care. The BCAP PDI is strengths-based, drawing on empowerment messages highlighting community mobilization against HIV.

The PDI approach taps into six critical elements of behavior change: knowledge, skill building, motivation, peer influence, social norms, and repetition [34, 47, 48]. When used with vulnerable populations, PDI capitalizes on the personal and community-minded transformative changes that often occur among such groups [49–52]. PDI is based on the Theory of Normative Regulation, which posits that the behaviors of individuals are shaped by social norms and amplified through their social groups [36]. When individuals appeal to peers’ behavior (e.g., HIV testing), their *own* behavioral commitments may be strengthened. Messages delivered by peers can be potent because peers can have more credibility than professionals [27, 53]. PDI is also cost-efficient, with much of the intervention conducted by participants.

#### Eligibility criteria and screening for eligibility

Study eligibility criteria are the same for initial seeds and peers with two exceptions. Study eligibility criteria are: age 18-60 years; sexually active (vaginal and/or anal sex) with at least one opposite sex partner within the previous year; reside in the larger HRA (but initial seeds must reside only in the core HRA); African American/Black or Latino/Hispanic race/ethnicity; comprehension of English or Spanish; unknown HIV status (for initial seeds only; peers may report previous HIV diagnosis); not actively psychotic; not a participant in the past local NHBS studies with HHR.

Peers present to the study with a coded recruitment coupon linking the recruiter to the recruit. Consenting recruits participate in a brief computerized screening interview to determine eligibility, including questions about their relationship with the recruiter. All screened participants are provided an incentive ($15) and *recruiters* are also compensated for each peer who presents to the study depending on peer eligibility ($5-25). Initial seeds are directly recruited by staff, as noted above, and are screened for eligibility using these same procedures. The next section describes procedures for participants who are HIV negative or do not know their HIV status at the time of screening, the main focus of the study, followed by a brief description of activities for those who enter the study with known HIV infection.

### BCAP PDI study activities

#### Seek and test activities

##### Peer education

**Fig. 3:**
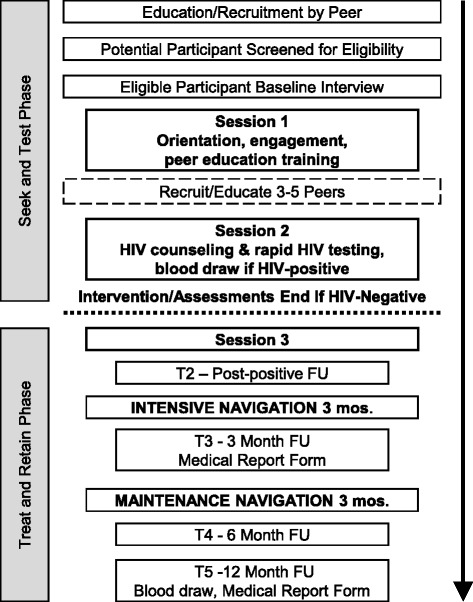
Schematic Representation of PDI/RDS and assessment sequence

For those in the intervention arm, the BCAP PDI begins when potential participants receive peer education on a small number of core messages from a study participant, along with a coded recruitment coupon. (See Fig. 3 for the sequence of intervention and assessment activities for the BCAP PDI intervention arm. Note that we show activities for both the Seek and Test and Treat and Retain phases in this figure for clarity, and because the Seek and Test activities create the basis for those conducted in the Treat and Retain phase.)

##### Baseline assessment

Participants complete a structured baseline interview administered via the Audio Computer-Assisted Interviewing format (ACASI) lasting 60-90 min. The baseline interview assesses socio-demographics, health, substance use, sexual behavior, HIV testing history, and peers’ experiences with HIV testing and risk behaviors. Baseline interviews mainly assessed the lifetime and past 6-month period and the battery is comprised mainly of a set of harmonized measures developed for the set of NIDA-funded STTR projects [54].

##### Introductory intervention session #1

Participants engage in an intervention session individually with a trained interventionist lasting 20-60 min, usually within two weeks. The goals of the session are to engage the participant and motivate him/her to conduct peer recruitment/education and continue in the study. Control arm participants are taught how to recruit peers for the study during this session, and intervention arm participants are also taught and motivated to *educate* their peers on the core messages during the course of recruitment. Participants receive 3 to 5 recruitment coupons. One coupon must be used for a female participant, as a “steering incentive” [55].

##### Recruitment period

Participants enter a two-week period where they may recruit and educate a small number of peers and provide them with recruitment coupons. Those peers then contact the study directly to be screened, and the participant continues in the study. Participants may decline to recruit peers and continue in the study.

##### Intervention session #2

Participants return for a second one-on-one intervention session two weeks after their initial session. During this session, CDC required pre-test counseling is provided to assess readiness and desire for testing. If participants decide to test, standard consent is obtained, followed by the OraQuick Advance Rapid HIV-1/2 Antibody Test with oral mucosal fluid. While the test is processing, subjects complete a computerized HIV prevention and risk-reduction program, called CARE for Prevention [56]. If the rapid HIV test is non-reactive, participants are counseled and provided referrals, and their involvement in the study is complete.

For reactive tests, a second OraQuick Advance Rapid HIV test is administered, similar to local health department protocols, to minimize false positive results on site [57]. An OraSure Western Blot confirmatory test is also conducted, with results available in 1-2 weeks. Participants are then asked to provide blood specimens for HIV viral load and CD4 testing. Because some newly diagnosed participants decline to provide blood at diagnosis, they may do so at a future contact if they so choose. Navigation (described below) may begin at this point for intervention arm participants who have serious barriers to engaging in future study activities, including meeting to receive confirmatory results, or the session may end after the blood specimen is provided and navigation may begin at Session 3.

##### Intervention session #3

Newly diagnosed participants are provided confirmatory test results and post-test counseling following CDC and local guidelines. Those in the control arm receive standard post-test counseling with referral to HIV health care and up to three phone reminders to attend the appointment. For intervention-arm participants, the session then turns to a discussion of the navigation component of the BCAP PDI. Participants receive compensation for each session ($30) and blood draws ($25), plus local round-trip public transportation.

#### Treat and retain phase activities

##### Navigation

Navigation is a flexible and individualized intervention approach to reduce disparities in care for low income or marginalized populations [58, 59]. In practice, navigation identifies and resolves the organizational, social, and individual barriers patients may experience to accessing these services [60]. In the BCAP PDI, navigation uses a strengths-based approach that draws on Motivational Interviewing techniques, guided by an Action Plan that is revisited over time. The aim of the Action Plan is to identify and resolve barriers to linkage to HIV care and support engagement in care. If there are no major barriers to care, the navigator can monitor and support engagement in care. If there are barriers (e.g., mental illness or substance abuse), navigation involves several options: communication with the primary care provider; screening and “fast track” referrals for substance use, mental illness, housing and other services; accompaniment to health care appointments; phone call reminders; and in-person meetings to support adaptation to the HIV diagnosis and linkage to care. Contacts are documented, and participants receive modest financial incentives for the first four navigation meetings to establish the working alliance during this critical period ($10 each).

#### Aspects of the PDI and assessments

##### Differences between the BCAP PDI and control arm

Intervention arm participants 1) receive peer education at the time of recruitment, which introduces them to the core messages, while controls do not receive peer education; 2) are trained to educate their own peers on the core messages that reinforce the core messages, while controls are trained only to recruit peers; and 3) the newly diagnosed receive patient navigation while controls receive treatment as usual (an HIV care appointment and follow-up phone calls), as shown in Fig. 1.

##### Network-based case finding

To increase access to the undiagnosed, participants who are newly diagnosed with HIV during the study are given the opportunity to recruit three additional peers, and encouraged to recruit sexual or injection drug-sharing partners if they choose. This is a form of network-based case finding [61, 62]. The newly diagnosed participant’s recruiter also receives coupons, as does one participant randomly selected from the same arm. This latter strategy is designed to protect the newly diagnosed participant’s confidentiality, as well as that of his/her recruiter, by masking the reason for receiving additional coupons.

##### Follow-up assessments

Participants in both arms are assessed at four time points: at Session 3, the time of diagnosis (the Time 2 [T2] assessment), and 3- (T3), 6- (T4), and 12-months post-baseline (T5). The T2, T3, and T4 interviews assess the prior quarter, and the T5 interview (12-month follow-up) assesses the prior 6 month period. The assessment includes measures on care engagement, satisfaction, understanding of HIV, and HIV medications prescribed, side effects, and adherence [63–65]. All assessments are conducted using ACASI and take approximately an hour. At T5, blood is drawn to measure CD4 count and HIV viral load; a Medical Report Form containing data on attendance at care appointments over the past year drawn from the medical record is also completed. Participants receive modest compensation for assessment activities ($25-$30).

#### Activities for those with previously diagnosed HIV infection at screening (“known positives”)

Participants with previous HIV diagnoses are hypothesized to be an important means of finding the undiagnosed [66]. Yet they also may be poorly engaged in HIV care, thereby in need of intervention. As noted in the section on Eligibility Criteria, the PDI/RDS study enrolls peers (but not initial seeds) with previously diagnosed HIV infection. At screening, the peer’s HIV status is confirmed with medical documentation. A baseline interview assesses the domains noted above, supplemented with data on HIV health status, engagement in care, experience with providers, and ART prescription and adherence [67]. Participants then engage in a single intervention session and are trained to recruit/educate (intervention arm) or recruit (control arm) peers, and their past-year HIV health care attendance patterns and satisfaction with care are evaluated to determine whether navigation to HIV care is required. Participants then have the opportunity to recruit 3-5 peers over a two-week period, and navigation to care is provided. At four months post baseline, a Medical Report Form capturing CD4, HIV viral load, and health care attendance over the past year from the medical record is completed.

#### Changes to procedures during implementation

Complex research studies often undergo refinements in their early stages. A number of changes were made to the initial study protocol over the first six months of implementation in response to high rates of ineligibility (> 40 %), insufficient rates of enrollment which stopped recruitment chains, low numbers of female participants, an imbalance between intervention and control arms, and low rates of HIV-infected participants in the study’s early phase of implementation. These study inefficiencies were reviewed by the study Principal and Co-Investigators, who made recommendations for adjustments. In response, changes to some eligibility criteria were instituted. Initially, participants were eligible if they had not been tested for HIV in the past year; were HIV negative or unknown status; were aged 18-*50* years; and resided only in the core seven zip code HRA. Further, there was initially no steering incentive for women. Third, the control intervention Session 1 was very brief (~5-10 min to orient participants and train them how to recruit peers, which was not sufficient for engagement). The changes to eligibility criteria reflected in the section above were designed to reduce ineligibility and improve study efficiency; steering incentives to boost the proportion of women in the sample; and increasing the duration of the control Session 1 is intended to improve engagement among the control arm participants. The final protocol described above will be implemented for > 75 % of the sample.

#### Data analysis

The hypothesis for the main Treat and Retain aim is that compared with participants in the control arm, PDI arm participants will be more likely to 1) attend an HIV clinic appointment sooner; 2) initiate ART earlier; 3) achieve viral load suppression; and 4) be retained in HIV care during the 12-month follow-up period. Logistic regression will be used to compare groups on viral load suppression. Since HIV viral load assay results range from <50 to >100,000 copies/ml, other generalized linear models will examine intervention impact on median viral load, log transformed viral load, and on viral load dichotomized at 1,000 or 10,000 copies/ml, which have been associated with greater HIV transmission risk [68–71]. Cox proportional hazards regression will compare groups on time to first HIV clinic appointment and initiation of ART if indicated. Frailty models [72, 73] will examine intervention effects on first HIV clinic appointment and initiation of ART with participants clustered by recruitment chain. Covariates for these analyses will include prior HIV testing and initial viral load.

#### Power analysis

We estimate 10 % of all participants will be newly diagnosed with HIV infection and assume an attrition rate of 15 % at the last follow-up among those with newly diagnosed HIV infection (*N* = 254). We estimate the smallest detectable difference in proportion between PDI and control arms given a desired power of 80 % and α = .05. Assuming 15-45% of control participants achieve HIV viral load suppression within 12 months, odds ratio of 2.07 are detectable. With an ICC as high as .20 due to clustering by 80 seeds, the effective sample size is *n* = 176 and odds ratios of 2.39 are detectable.

## Part 2: Seek and test phase

### Introduction

#### Study aims

**Fig. 4:**
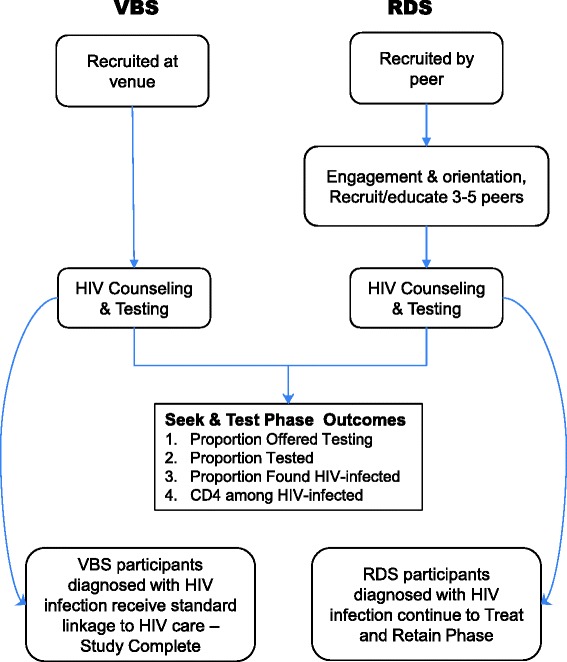
Seek and Test Phase

The aim of the Seek and Test Phase is to compare the relative yield and efficiency of RDS, described above, and venue-based sampling (VBS) to seek out and identify previously undiagnosed HIV infection among HHR, and to project the clinical impact, costs, and cost-effectiveness of these two strategies (as shown on Fig. 4). Although the NHBS uses both VBS and RDS methods, they have not yet been directly compared in terms of their efficacy and cost-effectiveness to identify undiagnosed HIV among HHR in the U. S. The present study addresses this gap in the literature by conducting VBS and RDS in the same geographical location, namely, the core HRA, and targeting the same underlying population of HHR.

#### Venue-based sampling (VBS)

VBS is a method to systematically recruit individuals in a target population who may be hard-to-reach, but who may be located and engaged in identifiable venues such as parks and hair salons. VBS has proven successful in identifying populations at high risk of HIV infection, mainly MSM [74–76], but also heterosexuals in the U.S. and globally [77, 78]. VBS starts by identifying days and times at which the target population gathers at specific venues, constructing a sampling frame of venue-day-time units (VDTs), randomly selecting and visiting VDTs (the primary sampling units), and systematically intercepting and collecting information from consenting members of the target population [74].

#### Hypotheses and endpoints

We hypothesize a higher proportion of newly diagnosed participants will be recruited by RDS than VBS, after controlling for socio-demographic differences between the samples. This is because RDS is designed to reach deep into hidden or wary populations, engaging the more isolated or vulnerable network members who may not be present in social venues [61]. Indeed, peers have credibility that can foster engagement with research studies more effectively than direct recruitment by research staff, even for those with multiple barriers to HIV testing [62].

## Methods

### Defining and identifying specific venues within the HRA

Once the core HRA had been defined, as we described above, we identified venues within it to establish the locations and times for sampling. A venue is defined as a public space where a threshold number of the target population may congregate or pass through [76]. Typically VBS studies target discrete venues such as churches, hair salons and barbershops, recreational centers, bars, city parks, parks or street corners outside public housing facilities, and grocery stores [76]. While members of the target population naturally congregate in these locations, these types of venues may require the permission of a manager or other gatekeeper before recruitment. Indeed, parks and public housing are regulated by the local authorities, and private venues can close during the study period. Thus targeting these types of venues is labor-intensive.

However, the population density, built environment, and population transportation dynamics of central Brooklyn suggested an alternate means of defining a venue, namely, as city blocks where the target population congregates or passes through. But since central Brooklyn is geographically large (70.82 sq. miles) and densely populated (2.6 million) [79], we needed to narrow the scope of potential venues to reduce redundancy and increase study efficiency.

To select specific venues for sampling during the study’s planning phase, we drew on Department of Urban Planning geographic data showing the location, land use (residential vs. commercial), and other attributes of every building in Brooklyn [80]. With this, we defined a “venue” as any city block within the core HRA with >70 % commercial land use. Study staff then reviewed a random sample from this sampling frame to qualitatively assess whether this approach would yield sufficient foot traffic for study recruitment. To supplement this block approach, we also included public parks and public housing projects as discrete venues for sampling.

Venue-day-time periods (VDTs) were chosen for each venue. VDTs were defined as four-hour blocks where a sufficient number of the target population passes through the venue. For each venue defined above, we included a morning, afternoon, and evening VDT for potential recruitment. This ultimately yielded a sampling frame of 394 block venues and 75 park and housing project venues available for random selection during the study, each with 3 associated time periods.

### Sampling VDTs

On a monthly basis, after excluding dates for holidays and related events, a sample is drawn from the sampling frame based on the number of planned recruitment events per month. This involves a two-stage sampling process: first a simple random sample of venues, then for each sampled venue, a random sample of associated time blocks. Sampling is done without replacement for each month, but previously selected VDTs are eligible for sampling in subsequent months. For each selected VDT, we also sample two alternate VDTs in the event that recruitment within the main VDT is infeasible due to safety concerns or low attendance. One week prior to the recruitment event, study staff members observe the selected venue to determine its suitability with respect to safety and other logistical concerns. Study staff also secures a location to conduct confidential study activities, or used a large mobile van. We will conduct up to 4 VBS events per month over 30 months for a total sample size of 400 participants enrolled.

### Eligibility criteria

The VBS eligibility criteria are the same as in the PDI/RDS study described above, with the following exceptions: VBS participants must reside in the core HRA (not the larger HRA), and those with previously diagnosed HIV are ineligible. Thus, the VBS eligibility criteria are: aged 18-60 years; sexually active (vaginal and/or anal sex) with at least one opposite sex partner within the previous year; reside in the core HRA’s seven zip codes; African American/Black or Latino/Hispanic race/ethnicity; comprehend English or Spanish; unknown or negative HIV status; not actively psychotic based on valid screening instrument.

### Primary outcome measure

The primary outcome is the yield and efficiency of VBS to identify undiagnosed HIV infection among HHR, which will be compared to rates in the RDS sample. HIV status is assessed with the OraQuick Advance Rapid HIV-1/2 Antibody Test using oral fluid and OraSure Western Blot for confirmatory testing using oral mucosal fluids.

### Description of study activities

#### Enrollment targets

The goal is to enroll 6 to 9 participants at each event. The field team is comprised of a supervisor, a HIV test counselor/interventionist (who deliver HIV test results), and 2-3 Research Associates. At least one staff member at every event is a trained phlebotomist. Team members include both males and females from diverse socio-economic and racial backgrounds who are knowledgeable about, comfortable with, and experienced with the local area, target population, and field recruitment methods. As in the BCAP PDI, the overarching counseling approach is Motivational Interviewing [46].

At the enrollment event, participants who cross a pre-specified recruitment line in the selected venue are approached by a staff member and recruited for the screening interview. All those who cross this line are counted to enumerate the venue size. Individuals who cross the line are asked if they would be willing to participate in a brief (15 min) health screening interview for a community health study, depending on staff availability. We estimate 50-75 % of participants who cross the line will be approached.

A brief screening interview determines study eligibility. Those who consent to participate but who are unable to complete the study activities at that time may complete activities at the study field site location within two weeks of recruitment.

Participants then engage in HIV pre-test counseling and receive a rapid OraQuick HIV test. During the 20-min processing period, the participant completes a structured baseline interview using ACASI, which measures socio-demographic and background factors, health, substance use, sexual behavior, and HIV testing history using a battery of measures collected across all NIDA STTR projects [81–83].

Participants with a non-reactive (negative) test result receive risk reduction counseling. Those with reactive test results are informed about the preliminarily positive result, and the need for confirmatory testing. A confirmatory test is conducted, and the interventionist initiates a brief discussion about this new diagnosis, individually tailored in light of the wide variety of participant reactions to HIV diagnoses [84]. Then the interventionist discusses the need for HIV primary care and makes an appointment for HIV primary care at a setting the participant finds convenient and acceptable. Third, the interventionist informs the participant that all those who test preliminarily positive are asked to give blood specimens, in order to document the initial health status of those with new diagnoses. In the final component of the post-test counseling session, participants are provided with the interventionist’s contact information, for support, referrals, or any other concerns and are scheduled for a return appointment for the confirmatory test result at the project field site two-weeks later. Participants receive compensation at an equivalent rate to the PDI/RDS study.

### Power analysis

For the Seek and Test study aim, 3000 HHR recruited by RDS are compared with 400 HHR recruited by VBS on the outcome of newly diagnosed HIV infection. Assuming 10 % of the RDS sample will have newly identified HIV infection, the study is powered to detect a difference in seek approaches of OR = 1.79 with 80 % power (α = .05). If the intraclass correlation (ICC) for previously undiagnosed HIV infection is as high as .20 (due to clustering of individuals within 80 recruitment chains), the effective sample size is as low as *n* = 361 in the RDS group, and OR = 2.25 is detected with 80 % power (α = .05).

### Analysis plan

We will examine differences in demographic characteristics between VBS and RDS participants and adjust for these when comparing VBS and RDS. The guiding hypothesis for the Seek and Test aim is the following: compared with VBS, and controlling for potential differences on key socio-demographic characteristics across the samples, participants recruited by RDS will be more likely to be newly diagnosed with HIV. This hypothesis is assessed using logistic regression analysis. Newly diagnosed HIV infection (Yes/No) is regressed on a dummy variable created to contrast participants recruited at venues with participants recruited by RDS. If the coefficient for this variable is positive and significant, we will conclude that RDS is a more effective approach for seeking HHR likely to have undiagnosed HIV infection. Covariates will include prior HIV testing.

## Discussion

The present study seeks to examine two approaches to uncovering undiagnosed HIV infection in an under-studied population: heterosexuals at high risk for HIV. Further, it tests components to link those with HIV-infection to HIV primary care with high retention, both newly diagnosed and those with previous HIV diagnoses. Study results will provide guidance on the most efficient and cost-effective means of uncovering this largely hidden and vulnerable population (by comparing RDS vs. VBS), and to link HIV-infected individuals to HIV care (by comparing the PDI arm vs. control). The ultimate aim of the present study is to provide an efficient, cost-effective, reproducible, and scalable sampling method and intervention approach to address the critical public health problem of undiagnosed HIV infection and delays in engagement in HIV care among HHR. This protocol provides background for other investigators interested in researching this population, which is challenging to define, reach, and engage.
